# Role of lactic acidosis as a mediator of sprint‐mediated nausea

**DOI:** 10.14814/phy2.14283

**Published:** 2019-11-13

**Authors:** Robert J. Merrells, Ashley J. Cripps, Paola T. Chivers, Paul A. Fournier

**Affiliations:** ^1^ School of Human Sciences Division Sport Science, Exercise and Health University of Western Australia Crawley Australia; ^2^ School of Health Sciences The University of Notre Dame Australia Fremantle Australia; ^3^ Institute for Health Research The University of Notre Dame Australia Fremantle Australia; ^4^ Exercise Medicine Research Institute & School of Medical and Health Sciences Edith Cowan University Joondalup Australia

**Keywords:** high intensity exercise, lactic acidosis, nausea, *nucleus tractus solitarii*, sprint

## Abstract

This study aims to determine whether there is a relationship between nausea level and lactic acidosis during recovery from sprinting. In all, 13 recreationally active males completed a 60 s bout of maximal intensity cycling. Prior to and for 45 min following exercise, blood pH, pCO_2_, and lactate levels were measured together with nausea. In response to sprinting, nausea, lactate, and H^+^ concentrations increased and remained elevated for at least 10 min (*p* < .001), whereas pCO_2_ increased only transiently (*p* < .001) before falling below pre‐exercise levels (*p* < .001), with all these variables returning toward pre‐exercise levels during recovery. Both measures of nausea adopted for analyses (nausea profile, NP; visual analogue scale, VAS), demonstrated significant repeated measures correlation (rmcorr) post‐exercise between nausea and plasma lactate (VAS and NPr_rm_> 0.595, *p* < .0001) and H^+^ concentrations (VAS and NPr_rm_> 0.689, *p* < .0001), but an inconsistent relationship with pCO_2_ (VAS r_rm_ = 0.250, *p* = .040; NP r_rm_ = 0.144, *p* = .248) and bicarbonate levels (VAS r_rm_ = −0.252, *p* = .095; NP r_rm_ = −0.397, *p* = .008). Linear mixed modeling was used to predict the trajectory of nausea over time, with both lactate and H^+^ concentrations found to be key predictors of nausea (*p* < .0001). In conclusion, this study reveals a strong positive relationship between nausea and both H^+^ and lactate concentrations during recovery from sprinting, a finding consistent with H^+^ and lactate being potential mediators of nausea post‐sprinting. However, as the timing of the recovery of both H^+^ and lactate was delayed, compared to that of nausea, further research is required to confirm these findings and investigate other potential mechanisms.

## Graphical abstract

The causes of nausea post‐sprinting are unknown. Given that plasma H^+^ and lactate levels change markedly in response to sprinting, the relationships between nausea level and both H^+^ and lactate levels were investigated for the first time. This study shows that changes in H^+^ and lactate levels offer a strong correlation with and significantly predict the severity of sprint‐mediated nausea.

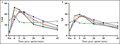

## 1 INTRODUCTION

The performance of a maximal sprint effort is integral not only to many sporting events but also to several high intensity training protocols, such as high intensity interval training, which are used extensively in team sport training and by the general public to improve health (Gibala, Little, MacDonald, & Hawley, 2012; Gillen et al., 2014). One drawback with engaging in high intensity exercise is that this form of activity is often associated with some unpleasant side effects such as pain, fatigue, nausea, and vomiting (Berning, Adams, Climstein, & Stamford, 2007; Burgomaster et al., 2008; Deighton, Barry, Connon, & Stensel, 2013; MacDougall et al., 1998; Richards et al., 2010; Rundqvist et al., 2017; Williams et al., 2013). Nausea, in particular, is a disagreeable experience that is biologically rooted in avoidance behavior to defend against noxious stimuli such as ingested toxins (Andrews & Horn, 2006; Oman, 2012; Treisman, 1977). Nausea is also associated with clinical conditions including but not limited to infection, gastrointestinal diseases, and anxiety disorders (Calza, Manfredi, & Chiodo, 2005; Quigley, Hasler, & Parkman, 2001; Singh, Yoon, & Kuo, 2016; Stern, Koch, & Andrews, 2011). Although there are no clearly defined categories of nausea, Stern et al. (2011) classify this condition according to the afferent pathway by which nausea is induced, specifically, the vestibular system, the area postrema, and the gut. In contrast, Oman (2012) divides nausea into its cause such as motion, toxins, or tastes and smells, while also including a feedback pathway by which a conditioned response may result.

The etiology of nausea associated with exercise has only been investigated in relation to endurance exercise (Brock‐Utne et al., 1988; Kondo et al., 2001; Samborski, Chmielarz‐Czarnocińska, & Grzymisławski, 2013; Stuempfle, Valentino, Hew‐Butler, Hecht, & Hoffman, 2016). There is evidence that, during endurance exercise, the redirection of blood flow from the gut to the exercising muscle may result in gut ischemia and mucosal damage, thereby increasing intestinal permeability (Costa, Snipe, Kitic, & Gibson, 2017; Oliveira, Burini, & Jeukendrup, 2014). These gastrointestinal perturbations, in turn, are associated with elevated plasma endotoxin levels and concomitant nausea (Brock‐Utne et al., 1988; Stuempfle et al., 2016), likely acting as a warning signal to cease the damaging behavior.

Despite the prevalence of nausea associated with sprinting, the mechanisms underlying sprint‐mediated nausea still remain to be investigated. Anecdotally, the nausea associated with high intensity exercise has been proposed to result from lactic acidosis (e.g., Marsh, 2016). Although there is no direct empirical evidence in support or not of this mechanism, a role for lactic acidosis as a mediator of nausea is supported by the observation that nausea is associated with acidosis of varying origins such as diabetic ketoacidosis (Anderson et al., 2013), renal failure (Jurovich, Wooldridge, & Force, 1997), and hyperchloremic acidosis (Sharma & Aggarwal, 2018). Moreover, a decrease in blood pH has previously been implicated in the activation of emesis (Kucharczyk, 1991).

Although high intensity sprinting to fatigue results in a marked rise in plasma lactate and H^+^ levels (Hermansen & Osnes, 1972), the relationship between these latter variables and nausea level post‐exercise remains to be investigated. One approach to provide some indirect evidence, albeit associative, of a possible causal relationship between nausea level and post‐exercise lactate and H^+^ levels, is to determine whether there is a relationship between nausea levels post‐sprint and lactic acidosis and whether the onset and recovery of nausea levels are preceded by changes in hydrogen ion or lactate concentration in blood. We hypothesized that the increased levels of blood hydrogen and/or lactate ion post‐exercise are closely associated with nausea level during recovery from a 60‐s maximal sprint.

## 2 METHODS

### 2.1 Participants

In all, 13 active healthy male volunteers, without a prior history of gastrointestinal problems or vertigo were recruited for the study. The procedures and potential risks of the study were explained to each participant before their written informed consents were obtained. All procedures were approved by the Human Research Ethics Committee of the University of Western Australia.

### 2.2 Experimental procedures

#### 2.2.1 Familiarization session

Six to eight days prior to the experimental session, all participants completed a familiarization session during which they were familiarized with the Central Discomfort Questionnaire (CDQ, see below) to measure nausea level. During this session, body mass, 10‐s peak anaerobic power, and $\dot{V}$O_2_ peak were also obtained. Participants were familiarized with the CDQ by completing it on five occasions, namely following the warm‐up, then at 0 and 10 min after both the 10‐s maximal power and $\dot{V}$O_2_ peak tests.

#### 2.2.2 Testing session

For 48 hr prior to the testing session, all participants were required to abstain from strenuous activity, and to fast overnight, consuming only water from 20:00 on the day prior to testing to ensure the stomach was empty of food. On arrival to the exercise laboratory at 07:30, the participants were weighed, then allowed a 5‐min self‐paced cycling warm‐up on an air‐braked cycle ergometer (Repco Exertech front access cycle ergometer, Repco Cycle Company, Huntingdale, Victoria, Australia), after which time they dismounted the ergometer. Then, a cutaneous vasodilating cream (Finalgon^®^, Boehringer Ingelheim, Atarmon, Australia) was applied and remained on the ear for 10 min prior to the collection of the initial sample. A sterile lancet (SoftTouch^®^, Roche Diagnostic Australia) was used to puncture the skin at the margin of the earlobe, and blood was collected in a 100 µl capillary tube, and immediately analyzed for pH, lactate (La^−^) and bicarbonate (${\text{HCO}}_{3}^{-}$) ion concentration, and partial pressure of carbon dioxide (pCO_2_) using a calibrated blood gas analyzer (ABL 625, Radiometer Copenhagen). Hydrogen ion concentration ([H^+^]) was calculated as 10^−pH^.

A pre‐exercise blood sample and CDQ scores were taken 5 min prior to the start of exercise, during which time the participants remained seated off the cycle ergometer until one minute prior to cycling. The participants then mounted the cycle ergometer and had their feet firmly strapped to the pedals. The exercise task involved a 60‐s Wingate‐style bout of maximal sprint cycling. The participants were instructed to cycle as quickly as they could, and informed that they were to achieve at least 95% of the peak power attained in the 10‐s peak anaerobic power test. All participants received the same verbal instructions, including a count down from three, and were required to start on “go.” Strong verbal encouragement was given throughout the entire test, and participants were not aware of the elapsed time. Peak power output (W) and total work (kJ) completed during the exercise bout were recorded.

A blood sample and CDQ assessments were completed immediately after exercise (0 min of recovery). Participants then moved from the cycle ergometer to a chair, and remained seated for the duration of the recovery, during which time further blood samples and CDQ measurements were collected at 5, 10, 20, 30, and 45 min of recovery.

#### 2.2.3 10‐s peak anaerobic power test

Prior to being subjected to peak anaerobic testing, participants were asked to warm‐up at a moderate intensity for 5 min on the same air‐braked cycle ergometer as that used for all testing (Repco Exertech front access bicycle ergometer, Repco Cycle Company, Huntingdale, Victoria). Prior to starting the test, participants stood on the pedals with the preferred leg at an angle of approximately 45° so as to elicit maximal power on the first stroke. Participants were allowed one practice start involving three seconds of maximal intensity pedaling followed by at least 2 min of rest before starting the 10‐s anaerobic power test. All participants received the same verbal instructions including a count down from three, and were required to start on “go.” Strong verbal encouragement was given throughout the entire test.

#### 2.2.4 $\dot{V}$O_2peak_ determination

$\dot{V}$O_2peak_ was determined on the same cycle ergometer as that used for all experimental trials. The $\dot{V}$O_2peak_ test consisted of a graded exercise test commencing at an intensity of 50 W at a self‐selected cadence. Thereafter, the intensity was increased by 30 W every 4 min until participants could no longer maintain the required power output. All expired air was collected through a mouthpiece connected to a Hans‐Rudolf valve attached via Collins tubing to an online gas analysis system comprised of a Morgan turbine ventilometer (Morgan, Model 096, Kent, England) and an O_2_ and CO_2_ gas analyzers (Ametek gas analyzers SOV/S3A and COV CD3A, respectively, Pittsburg, PA, USA).

#### 2.2.5 Central discomfort questionnaire

All participants were administered a central discomfort questionnaire (CDQ) composed of the nausea visual analogue scale (VAS), the “Nausea Profile” (NP) questionnaire (Muth, Stern, Thayer, & Koch, 1996), and selected subscales of the modified “Profile of Mood States” (POMS) questionnaire (Grove & Prapavessis, 1992). Nausea was assessed using the VAS, with nausea intensity rated on a continuous 100 mm line (Muth et al., 1996). Participants were asked to draw a single mark through the line at the point corresponding to the intensity of their nausea, the ends of the line were anchored with the descriptors no nausea on the left and extreme nausea on the right. The distance from the start of the line to the mark was measured to the nearest millimeter and reported as score out of 100. The NP questionnaire (Muth et al., 1996) includes somatic (six questions), gastrointestinal (five questions), and emotional distress (six questions) subscales. The POMS‐derived questions were added to the VAS and NP questionnaire to constitute the CDQ primarily to deceive the participants about the true purpose of the study. Participants were informed that the study was concerned primarily with measuring feelings of fatigue and discomfort post‐exercise, thus explaining the inclusion of POMS subscales. Two subscales were taken from the POMS questionnaire, namely the confusion (six questions) and fatigue (five questions). All answers were ranked on a scale of 0–9 with 0 indicating “not at all” and 9 indicating “severely” in the case of the NP questions or “extremely’ for POMS‐related questions. The sum of all scores was expressed as a percentage of the maximum attainable score for each of the NP subscale and NP as a whole. The NP was used in addition to the VAS as it allows for the evaluation of the three dimensions of nausea, providing further insight into the individual experience of nausea following high intensity exercise. Overall, the CDQ was composed of 26 questions and took approximately 70 s to complete.

Prior to exercise, the participants could read and answer the questions themselves. However, following exercise and during all experimental trials, with the exception of the VAS, which the participants always completed, the questions were read to the participants and verbal answers recorded. On all occasions, participants were instructed to answer the questions truthfully and as quickly as possible.

### 2.3 Statistical analyses

The statistical software SAS^®^ 9.4 (Institute, 2018) was used for all descriptive statistics and linear mixed modelling (LMM) using the Proc Mixed procedure. All variables were determined to be normal using a Shapiro–Wilks test and then described using mean and standard deviation (*M* (*SD*)).

Comparisons of pre‐exercise to post‐exercise values were performed for all variables using LMMs. The covariance parameters were estimated using the residual (restricted) maximum likelihood method, and a standard (variance components) variance structure for the random effect. Significance was set at *p* < .05, and all *p* values were reported following a post hoc Bonferroni adjustment. To assess the changes with time resulting from exercise, the VAS, NP, and its subscales (somatic, gastric, and emotional distress), [H^+^], La^−^ concentration, ${\text{HCO}}_{3}^{-}$ concentration, and pCO_2_ were treated as dependent variables.

A top‐down strategy, as outlined by West, Welch & Galecki (2014), was used to predict the occurrence and resolution of nausea using the VAS and NP including the somatic, gastric, and emotional subscales as dependent variables. The covariates, [H^+^], La^−^ concentration, pCO_2,_ and ${\text{HCO}}_{3}^{-}$ levels were added to the model as fixed effects. The type 2 tests of fixed effects were used to determine the significance of the variables, and all other LMM structures were as described above. Significance was set at *p* < .05, and where the calculated probability of the type 2 fixed effect was found to be greater than 0.05, the variable was discarded from the model, and the model was reassessed with the remaining variables. This procedure was continued until all variables were found to be less than 0.1 whence forth the Bayes information criteria (BIC) was compared to the previous model and the model with the lesser value was reported. Additionally, each covariate was assessed to determine its individual significance for the prediction of nausea, and for VAS and NP all significant covariates were reported. A residual diagnostics plot was used to investigate the assumptions of normality, and variance of the reported models did not violate this assumption.

To control for the repeated sampling of participants, all correlations were completed using the repeated measures correlation (rmcorr) method of Bakdash and Marusich (2017) using the rmcorr package (version 0.2.0) (Bakdash & Marusich, 2017) in R statistical software version 3.4.1 (R Core Team, 2017). All repeated measures correlations were interpreted according to the method of Cohen (1988), with statistical significance set at *p* < .05. Correlation (r) from the rmcorr analysis is denoted as r_rm_.

## 3 RESULTS

### 3.1 Participants

In all, 13 recreationally active healthy males aged 18.7–33.5 years participated in the study. Their descriptive characteristics, provided in Table 1, show that while they were active neither their aerobic fitness nor peak values indicate that they were highly trained. Comparison of the peak power attained in the 10 versus 60‐s sprints indicates that there was no difference between sprints, *t *(12) = 0.91, *p* = .38.

**Table 1:** Participant characteristics

Variable	Mean ± *SD*
Age (yr)	24.4 ± 4.5
Body mass (kg)	78.6 ± 12.6
$\dot{V}$O_2 max_ (ml/kg/min)	54.9 ± 5.1
10‐s peak power (W)	1,134 ± 257
60‐s peak power (W)	1,085 ± 239
60‐s peak power (W/kg)	13.88 ± 2.77
60‐s total Work (kJ/kg)	32.3 ± 5.7

(*n* = 13).

### 3.2 Effect of a 60‐s sprint on nausea profile scores and nausea visual analogue scales

**Figure 1:**
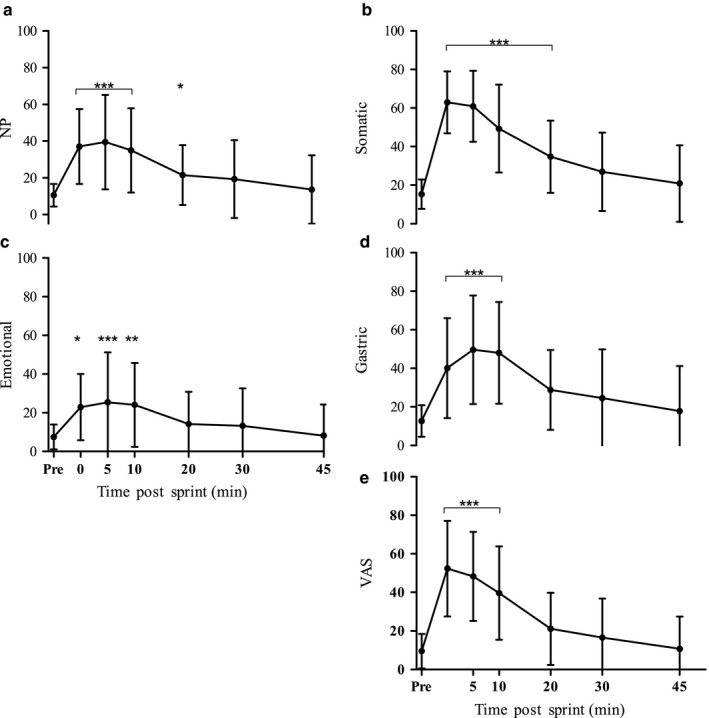
Effect of a 60‐s maximal sprint on nausea level. (a) Nausea profile, (b) Somatic distress, (c) Gastric distress, (d) Emotional distress, (e) VAS. Values are means ± *SD*; *n* = 13. *Indicates significant difference from pre‐exercise level *p < *.*05, **p < *.*01, ***p < *.*001

In response to exercise, NP levels increased significantly (*p* < .0001) to reach a maximum score of 39.5 (25.7) at 5 min of recovery (Figure 1a). During recovery, NP levels gradually decreased, and were no longer statistically higher than pre‐exercise values by 30 min post‐exercise (*p* = .24; Figure 1a). Each of the three NP subscale scores (somatic, gastric, and emotional distress) increased in response to the 60‐s maximal sprint, with somatic distress level peaking at 0 min with a score of 62.9 (16.0) (*p* < .001), while both gastric and emotional distress levels peaked at 5 min, reaching levels of 49.6 (28.2) (*p* < .001) and 25.5 (25.8) (*p* < .001), respectively (Figure 1b–d). As recovery progressed, each of these three NP subscale scores fell gradually, and were no longer different from pre‐exercise levels by 30 min of recovery for the somatic and gastric subscale, while the emotional subscale was no longer different by 20 min (*p > *.05).

In response to exercise, VAS increased significantly (*p* < .0001) to reach a maximum of 52.4 (24.8) at 0 min (Figure 1e). VAS levels then decreased during recovery and were no longer statistically higher than pre‐exercise values by 20 min post‐exercise (*p* = .30).

### 3.3 Effect of a 60‐s maximal sprint on blood variables

**Figure 2:**
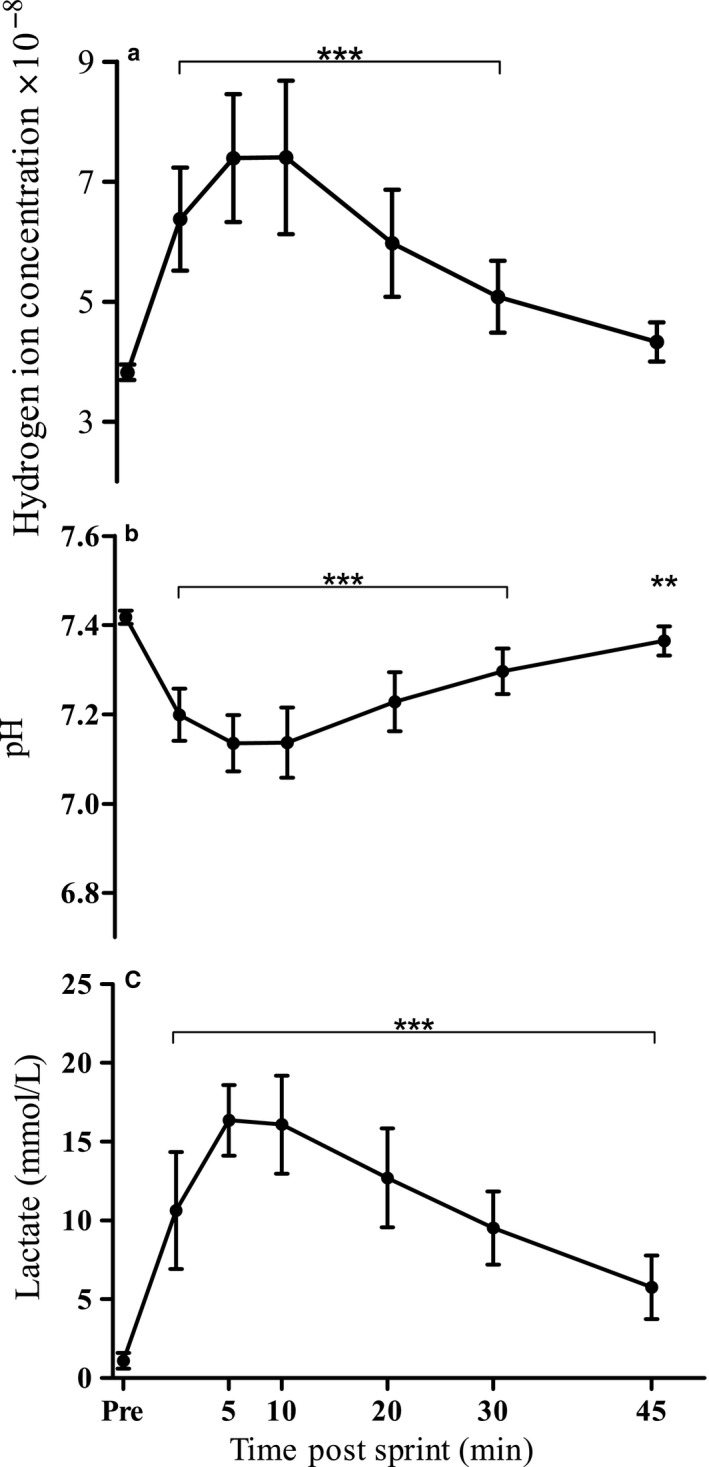
Effect of a 60‐s maximal sprint on blood (a) hydrogen ion concentrations, (b) pH, and (c) lactate levels. Values are means ± *SD*; *n* = 13. *Indicates significant difference from pre‐exercise level *p < .05, **p < .01, ***p < .001

Immediately following the 60‐s maximal sprint, pH decreased significantly to 7.20 (0.06) (*p* < .001) and continued to fall thereafter, reaching the lowest levels, 7.14 (0.08) (*p* < .001), at 10 min of recovery (Figure 2a). This corresponded to a maximum H^+^ concentration of 7.40 (1.27) × 10^−8^ mmol/l (*p* < .001; Figure 2b). As recovery progressed, pH gradually increased and remained lower than pre‐exercise value for the duration of the recovery period (*p* = .007). H^+^ concentration, however, decreased during recover and was no longer higher than pre‐exercise values at 45 min post‐exercise (*p* = .23).

Immediately following exercise, blood lactate increased significantly to reach a maximum level of 16.4 (2.2) mmol/L at 5 min of recovery (*p* < .001; Figure 2c). Lactate levels remained high at 10 min before gradually decreasing but remained statistically higher than the pre‐exercise value for the duration of the recovery period (*p* = .0001).

**Figure 3:**
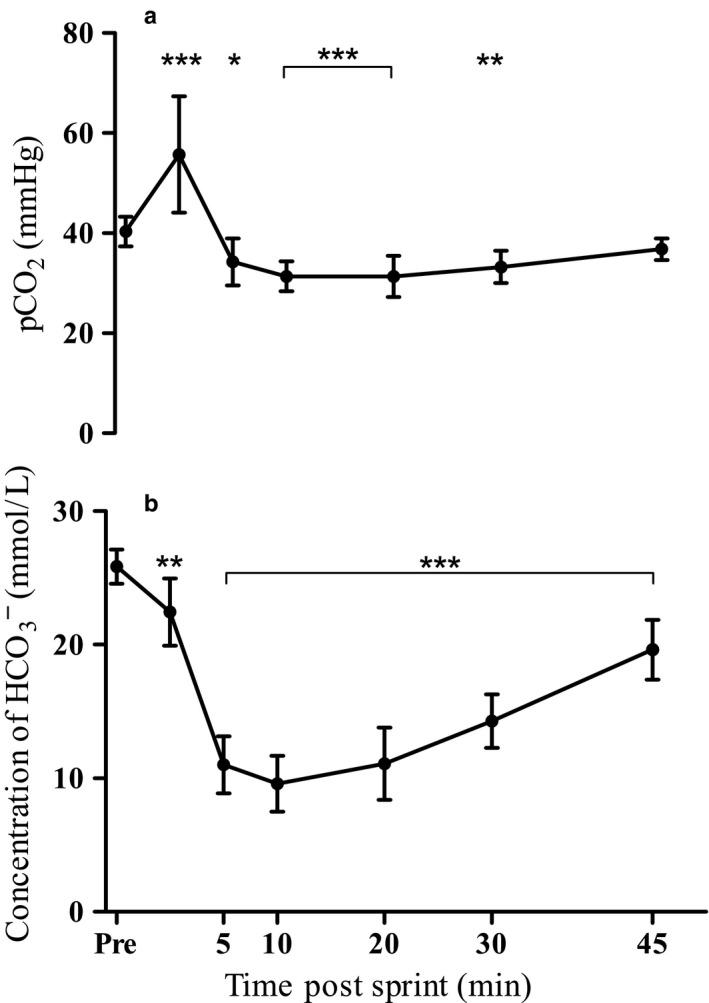
Effect of a 60‐s maximal sprint on blood (a) pCO_2_ and (b) ${\text{HCO}}_{3}^{-}$ levels. Values are means ± *SD*; *n* = 13. *Indicates difference from pre‐exercise * *p* < .05, ** *p* < .01, *** *p* < .001

Immediately following exercise, blood pCO_2_ increased significantly to levels of 55.7 (11.6) mmHg (*p* < .001) before rapidly decreasing to lower than pre‐exercise levels of 34.3 (4.1) mmHg at 5 min of recovery (*p* = .045). Thereafter, pCO_2_ decreased further, reaching minimum values of 31.4 (3.0) mmHg at 10 min of recovery (*p* < .001), after which pCO_2_ remained at stable levels until 20 min before gradually increasing to return to pre‐exercise levels at 45 min of recovery (*p* = .58; Figure 3a).

Bicarbonate ion concentrations decreased significantly following the 60‐s maximal sprint (*p* < .05), and remained significantly lower than pre‐exercise levels for the duration of the recovery period (see Figure 3b).

### 3.4 Relationship between nausea and blood [H^+^], lactate pCO_2_, ${\text{HCO}}_{3}^{-}$, and pO_2_

Repeated measures correlation analysis indicated that both blood H^+^ and lactate concentrations correlated positively with VAS, NP and each of the NP subscales post‐exercise, whereas pH exhibited a negative correlation with these variables (Table 2). Of the NP subscale scores, the emotional distress subscale had the lowest correlation with each of the blood variables (see Table 2).

**Table 2:** Repeated measures correlation between hydrogen ion concentration, pH, blood lactate, bicarbonate, and nausea levels, VAS, NP, and NP subscales

	r_rm_	95% CI		*p*	*df*
		Lower	Upper		
[H^+^]					
VAS	0.689	0.538	0.797	<0.0001	67
NP	0.711	0.660	0.813	<0.0001	66
Somatic	0.733	0.598	0.791	<0.0001	66
Gastric	0.731	0.595	0.827	<0.0001	66
Emotional	0.602	0.422	0.737	<0.0001	66
pH					
VAS	−0.683	−0.793	−0.530	<0.0001	67
NP	−0.71	−0.812	−0.565	<0.0001	66
Somatic	−0.752	−0.841	−0.624	<0.0001	66
Gastric	−0.721	−0.820	−0.580	<0.0001	66
Emotional	−0.594	−0.731	−0.411	<0.0001	66
Lactate					
VAS	0.595	0.415	0.723	<0.0001	68
NP	0.638	0.470	0.762	<0.0001	67
Somatic	0.680	0.526	0.791	<0.0001	67
Gastric	0.703	0.557	0.807	<0.0001	67
Emotional	0.534	0.338	0.687	<0.0001	67
pCO_2_					
VAS	0.250	0.008	0 464	0.040	66
NP	0.144	−0.103	0.375	0.248	65
[${\text{HCO}}_{3}^{-}$]					
VAS	−0.252	−0.513	0.052	0.095	43
NP	−0.397	−0.626	−0.106	0.008	42

Note

r_rm_ denotes repeated measures correlation coefficient, [H^+^] denotes hydrogen ion concentration, pCO_2_ denotes partial pressure of carbon dioxide and [${\text{HCO}}_{3}^{-}$] denotes bicarbonate ion concentration.

Blood pCO_2_ showed a medium positive correlation with VAS (*p* = .040), but was not significantly correlated with NP (*p* = .248), or any of the NP subscales (*p* > .05; individual subscale results not shown). NP, but not VAS, showed a significant moderate negative correlation with ${\text{HCO}}_{3}^{-}$ (Table 2).

The model estimates and statistic results calculated by LMM for VAS, NP, and NP subscales are depicted in Table 3. Prediction equations may be constructed by entering the values in Table 3 into the linear regression equation (Equation 1) as shown in Equation 1a. The LMM showed that [H^+^], lactate, and pCO_2_ were each significant factors for both VAS and NP on their own and in combination (Table 3). When the lactate concentration and pCO_2_ were entered in the model in combination (as in Equation 1a), the equation had the lowest Bayesian information criterion (BIC), thus better predictive ability. However, [H^+^] had the lowest BIC of each of the covariates on their own (see Table 3).

(1) $$\begin{array}{cc} \text{Predicted dependent variable} & =\text{intercept}\;+\;\beta \;\text{estimate}\;\times \;\text{covariate}\;1\;\\ & \;+\;\beta \;\text{estimate}\;\times \;\text{covariate}\;2 \end{array}$$

**Table 3:** VAS, nausea profile, and subscale mixed models: estimates of fixed effects for parameters

Model number	Dependent variable	Parameter	Intercept and Estimate (β)	*SE*	95% CI		*p* value	BIC
					Lower	Upper		
1	VAS	Intercept	−30.58	7.99	−47.99	−13.17	0.0024	695.2
		[H^+^]	10.18	1.26	7.67	12.69	<0.0001	
2	VAS	Intercept	4.64	5.36	−7.03	16.31	0.4032	729.2
		Lactate	2.39	0.38	1.63	3.16	<0.0001	
3	VAS	Intercept	7.00	10.97	−16.90	30.91	0.5353	733.7
		CO_2_	0.55	0.27	0.01	1.09	0.0467	
4	VAS^*^	Intercept	−58.05	10.57	−81.08	−35.01	0.0001	676.0
		[H^+^]	10.53	1.16	8.21	12.84	<0.0001	
		CO_2_	0.68	0.18	0.31	1.05	0.0004	
5	VAS	Intercept	−44.47	9.95	−66.15	−22.78	0.0008	654.4
		Lactate	2.93	0.33	2.27	3.59	<0.0001	
		CO_2_	1.15	0.20	0.75	1.55	<0.0001	
6	NP	Intercept	−15.64	6.41	−29.60	−1.68	0.0311	633.7
		[H^+^]	7.05	0.84	5.37	8.73	<0.0001	
						
7	NP	Intercept	8.28	5.45	−3.59	20.14	0.1544	663.9
		Lactate	1.67	0.25	1.18	2.16	<0.0001	
8	NP	Intercept	15.75	8.42	−2.58	34.09	0.0858	677.4
		CO_2_	0.23	0.19	−0.14	0.60	0.222	
10	NP^†^	Intercept	−28.56	8.11	−46.23	−10.89	0.0042	622.6
		[H^+^]	7.26	0.82	5.62	8.89	<0.0001	
		CO_2_	0.32	0.13	0.06	0.57	0.0158	
11	NP	Intercept	−18.67	8.01	−36.12	−1.23	0.0379	606.5
		Lactate	1.94	0.23	1.47	2.41	<0.0001	
		CO_2_	0.64	0.14	0.37	0.92	<0.0001	
12	Somatic	Intercept	−35.88	8.58	−54.57	−17.19	0.0013	622.1
		Lactate	3.05	0.27	2.51	3.59	<0.0001	
		CO_2_	1.14	0.16	0.82	1.46	<0.0001	
13	Gastric	Intercept	−19.62	7.66	−36.31	−2.93	0.0249	660.2
		[H^+^]	8.78	0.99	6.80	10.77	<0.0001	
14	Emotional	Intercept	−12.04	6.02	−25.17	1.08	0.07	623.6
		[H^+^]	4.97	0.79	3.40	6.54	<0.0001	

Note

[H^+^] denotes hydrogen ion concentration, CO_2_ denotes partial pressure of carbon dioxide, and [${\text{HCO}}_{3}^{-}$] denotes bicarbonate ion concentration. All [H+] estimates are ×10^8^. The model with the lowest BIC, according to the top down method of West and colleagues (West et al., 2014) is denoted by * for VAS and † for NP. Only the model with the lowest BIC according to the aforementioned strategy is shown for the somatic, gastric, and emotional distress subscales.

For example

(1a) $$\begin{array}{cc} \text{VAS}\;\left(\text{predicted}\right)\; & =-44.47\;+\;2.93\;\times \;\text{lactate concentration}\\ & \;+\;1.15\;\times \;p{\text{CO}}_{2} \end{array}$$

**Figure 4:**
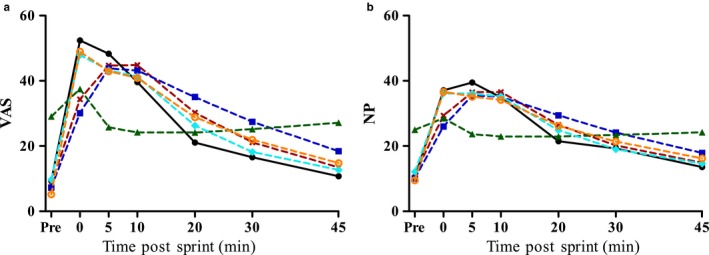
Actual mean nausea scores (*n* = 13) versus nausea scores as measured by VAS (a) and the NP (b) predicted by the linear mixed models. Closed lines and ● denote actual scores, dotted lines denote calculated nausea values using the prediction equation as outlined in Equation 1a and the values in Table 3 above. The individual covariate models are [H+] denoted by (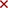), La^−^ (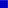), CO_2_ (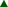), [H+] with pCO_2_(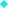) and La^−^ with pCO_2_ (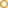), respectively. All covariates were significant in all models (*p* < .05), with the exception of pCO_2_ in the prediction equation for NP (*p* = .222) as shown in Table 3 above

The good fit of the models was supported by residual diagnostics, which followed an approximate normal distribution. Comparisons of the predicted nausea response and actual scores showed a good fit (Figure 4). The prediction equations for all models had a tendency to underestimate both the peak value and rate of recovery, resulting in lower predicted values at time 0 and 5 min, whereas at 20 min post‐exercise the predicted values exceed the actual values (Figure 4a and b, figures for somatic gastric and emotional subscales not shown). Although pCO_2_ (Figure 3) did not follow the same trend as nausea (pCO_2_ increased rapidly to be significantly higher than pre‐exercise values at 0 min before rapidly decreasing to below pre‐exercise pCO_2_ at 5 min of recovery), it was found to be a significant determinant of nausea, but as expected had the highest BIC, and thus the lowest predictive ability of all models.

## 4 DISCUSSION

The aim of this study was to provide some evidence of a causal relationship between exercise‐mediated nausea and both blood [H^+^] and lactate levels. This was achieved by examining whether there is a temporal relationship between the severity of nausea symptoms and the levels of H^+^ and lactate in blood post‐exercise. The findings of a strong positive correlation between nausea level and both [H^+^] and blood lactate concentrations together with [H^+^] and lactate levels being significant factors in our linear mixed model to predict nausea level are consistent with the existence of a causal relationship between the severity of nausea and both [H^+^] and lactate levels post‐sprinting. While establishing an association is the first step toward establishing a causal link, it must be understood that a correlation between variables does not necessarily entail causality. For this reason, further work is required to support our interpretation.

Since the temporal relationship between nausea level and blood [H^+^] or lactate concentration has not been described previously in the literature, this limits our capacity to compare our findings with those of others. To the best of our knowledge, this is the first study to examine the temporal pattern of change in nausea level post‐high intensity exercise, with our findings showing a comparable pattern of change irrespective of the method adopted to measure nausea level. Although some studies have reported, but not measured, nausea level after high intensity exercise (Berning et al., 2007; Burgomaster et al., 2008; Deighton et al., 2013; MacDougall et al., 1998; Richards et al., 2010; Rundqvist et al., 2017), only two studies have measured nausea level following high intensity exercise, namely that of Williams and colleagues (Williams et al., 2013), using 4× 30 s Wingate tests, and that of Chong and colleagues (Chong, Guelfi, & Fournier, 2011), using a 30‐s maximal sprint, with the latter also measuring pH post‐exercise. Of note, both studies reported smaller increases in nausea level compared to our study, in absolute terms and relative to their reported changes in lactate or pH.

As mentioned above, the presence of a significant positive relationship between [H^+^] and nausea level together with [H^+^] being a significant factor in our linear mixed model to predict nausea is a finding consistent with the existence of a causal relationship between [H^+^] and nausea level post‐sprinting. This interpretation, however, is challenged by our findings that nausea level peaked earlier rather than after peak blood [H^+^]. Indeed, if nausea were to be mediated by changes in [H^+^] that are centrally detected by specific regions of the brain, such as the chemoreceptor trigger zone (Kucharczyk et al., 1991), one would expect the peak in blood [H^+^] to precede the nausea peak. However, since changes in muscle [H^+^] may activate nociception pathways via peripheral acid‐sensing ion channels (ASICs) (Immke & McCleskey, 2001; Yagi, Wenk, Naves, & McCleskey, 2006) and induce pain leading to muscle fatigue (Gregory, Brito, Fusaro, & Sluka, 2016; Law et al., 2008; Naves & McCleskey, 2005), maybe such a mechanism also contributes to the early peak in nausea level observed here since intramuscular [H^+^] and lactate normally reach maximum levels at the onset of recovery and earlier than maximum blood [H^+^] and lactate levels (Hermansen & Osnes, 1972).

The presence of a significant positive relationship between blood lactate concentrations and nausea level together with lactate concentration being a significant factor in our linear mixed model to predict nausea is also a finding consistent with the existence of a causal relationship between lactate concentration and nausea level post‐sprinting. These findings, therefore, do not exclude the possibility that it is lactate rather than [H^+^], or both, that mediates the increase in nausea post‐sprinting. In support of this view, nausea has been reported to be a symptom of hyperlactatemia (Calza et al., 2005) and has been observed to occur at low exercise intensity with a concomitant abnormal increase in lactic acidosis resulting from a mitochondrial disorder (Schrank, Schoser, Klopstock, Schneiderat, & Horvath, 2017). However, as was the case with [H^+^], a role for lactate as a mediator of nausea post‐exercise is challenged by the observation that nausea reached its peak level prior to maximal blood lactate concentration. Furthermore, while nausea was no longer increased above resting levels at 30 min post‐exercise, lactate remained higher for the duration of the recovery period, thus also challenging the relationship between nausea and lactate.

The mechanisms whereby changes in blood [H^+^] mediate nausea remain to be determined. Changes in [H^+^] may be detected within the *nucleus tractus solitarii* (NTS) (Lin, Jones, & Talman, 2018; Nattie & Li, 2012) or other areas of the central/peripheral nervous system via H^+^ interaction with chemoreceptors such as H^+^‐sensing G‐protein‐coupled receptors or the ASICs (Levin & Buck, 2014; Tresguerres, Buck, & Levin, 2010). Of note, ASICs are found throughout the body including the NTS (Huda et al., 2012) and also have afferent terminals in the NTS (Boscan, Pickering, & Paton, 2002). The NTS is fundamental in the processing of nausea signals as it is in this area that the peripheral afferent signals from the three key areas of nausea generation (the vagus, *area postrema*, and vestibular apparatus) converge (Babic, Browning, Mcmahon, Van, & Gilad, 2014). However, the possibility of a link between the stimulation of ASICs or other H^+^‐sensing receptors and nausea remains to be determined.

An alternative pathway by which acidosis may induce nausea is by altering the electrical activity and therefore wave propagation along the gastrointestinal tract. The induction of a respiratory acidosis or metabolic acidosis at a level similar (PaCO_2_ 61.2 mmHg and pH 7.25, respectively) to that reported in this study has previously been shown to increase gastric arrhythmia in anesthetized pigs (Tournadre, Allaouchiche, Malbert, & Chassard, 2000). Gastric dysrhythmia is associated with the development of nausea in humans (Koch, 2014). However, whether gastric dysrhythmia is a marker or cause of nausea remains unknown (Stern et al., 2011). Of note, however, gastric dysrhythmia can result in decreased gastric motility (O’Grady et al., 2014), which has been observed in high intensity exercise (Leiper, Broad, & Maughan, 2001), and is implicated in the development of nausea during endurance exercise (Oliveira & Burini, 2009).

The mechanisms whereby high lactate levels may mediate nausea remain to be elucidated. Since the response of ASICs to H^+^ is enhanced by the presence of lactate (Immke & McCleskey, 2001), and assuming that ASICs stimulation plays a role in mediating nausea, maybe the high lactate levels post‐sprinting stimulate nausea indirectly by enhancing the ASICs responses to H^+^. Also, given that lactate is more than simply an end‐product of glycolysis, with lactate being not only the primary fuel oxidized by neurons but also an important signaling molecule that plays some role in the consolidation of memory avoidance learning (Magistretti & Allaman, 2018), it is possible that this molecule is a mediator of nausea under conditions associated with excessive lactate levels. Such a postulated role for lactate is consistent with the observation that the neurons of the *area postrema*, an important emetic center, are in direct contact with blood and thus in a position to respond to acute changes in plasma lactate levels (Price, Hoyda, & Ferguson, 2008).

The mismatch between the timing of the peak in nausea level and both the peak in H^+^ and lactate levels raises the issue of whether these findings could also be explained on the grounds that the earlier peak in nausea level may be mediated, at least in part, by the early transient rise in blood pCO_2_ levels rather than only by lactate or H^+^ ions. Although there was no significant repeated measures correlation between the pCO_2_ and nausea level post‐exercise, it is noteworthy that pCO_2_ increased to peak levels immediately following exercise and returned within 5 min to basal levels. Also, that pCO_2_ might be implicated in nausea is suggested by our mixed linear model indicating that nausea level post‐sprinting is best predicted by a model combining both CO_2_ and either H^+^ or lactate rather than H^+^ or lactate alone. A few mechanisms may be proposed to explain how the early transient rise in pCO_2_, most probably brought about by a H^+^‐mediated protonation of ${\text{HCO}}_{3}^{-}$, may be involved in the development of nausea at the onset of recovery. One possibility is that the increased diffusion of CO_2_ from the blood into neuronal cells may result in a CO_2_‐mediated acidification of the cerebrospinal fluid (Maren, 2017). Whether such a predicted fall in pH could in turn trigger nausea remains to be determined. Another possibility relates to the observation that there are chemosensitive neurons within the NTS that respond to changes in pCO_2_ via activation of CO_2_‐sensing proteins (Dean, Bayliss, Erickson, Lawing, & Millhorn, 1990; Putnam, Filosa, & Ritucci, 2004). It is important to note, however, that our interpretation that CO_2_ is a mediator of nausea is challenged by the observation that nausea is only “rarely” reported during exposure to a 35% CO_2_ inhalation challenge test for anxiety (Schmidt, 1999).

Why should nausea occur following high intensity exercise? Exercise, in moderation, has many health benefits (Myers et al., 2015); however, a maximal sprint effort to near exhaustion is associated with a rapid disruption to homeostasis. In only 60 s, blood pH, lactate, and bicarbonate concentrations change from normal to values that are clinically significant in emergency medicine (Kraut & Madias, 2014; Mizock, 1989). In this context, exercise‐induced diversion from normal [H^+^] would be considered threatening to survival if it were to be sustained as maintenance of the bodies [H^+^] is tightly controlled because extreme changes in [H^+^] may denature proteins, activate or inhibit ion channels and receptors (Immke & McCleskey, 2001), affect metabolic processes (McMahon & Jenkins, 2002), and alter nerve transmission (Huda, McCrimmon, & Martina, 2013). On these grounds, it then seems plausible that the nausea, malaise, disorientation, light‐headedness, and fatigue associated with a maximal sprint effort constitute protective mechanisms against excessive disruption of homeostasis and provide strong signals for a prolonged period of rest to allow recovery to take place before activity is resumed.

The main limitation with this study is that causality between nausea level and both H^+^ and lactate levels post‐sprinting is inferred from the close association between these variables. Although such associations do not necessarily imply causality, the existence of a correlation between variables generally constitutes the first step toward establishing the presence of causal links between variables. In other words, although statistical analyses may indicate that a relationship is present between nausea level and both H^+^ and lactate levels, this relationship should not be interpreted as being necessarily causal, nor should we rely solely on such statistical analyses to exclude the involvement of variables that show poor associations (e.g., proposed role of pCO_2_ in the onset of nausea described above). Alternatively, the etiology of nausea may involve mechanisms not examined in this study. Nausea is a complex phenomenon and research into its etiology has been directed in many areas such as the role of vagal nerve activity, neurotransmitters, catecholamines, and changes to gastrointestinal blood flow and function (Stern et al., 2011). Future intervention‐based studies should be undertaken to provide further evidence for a causal role of [H^+^], lactate and maybe pCO_2_ in sprint‐mediated nausea, as well as explore other potential mechanisms such as a gastrointestinal blood flow decrease which has previously been observed following exercise (Qamar & Read, 1987), coinciding with the rise in nausea seen in the current study. The results of this study should also be considered with respect to the small sample size, however, statistically significant results suggest that Type II error is unlikely, and the study was adequately powered. A larger sample in future studies may improve any uncertainty associated with Type I errors.

## 5 CONCLUSION

This study provides the first evidence of a temporal association between nausea level and both [H^+^] and lactate levels. Specifically, this study shows that both [H^+^] and lactate levels exhibit a strong significant relationship with, and good ability to predict, nausea level following a 60‐s maximal sprint effort. Although these findings are consistent with the presence of a causal relationship between these variables, further research is required to ascertain whether this is the case. These are important issues to address as improving our understanding of the etiology of sprint‐mediated nausea may lead to interventions to manage the nausea associated with this type of exercise and improve our general understanding of the biology of nausea.

## CONFLICT OF INTEREST

No conflicts of interest, financial or otherwise, are declared by the authors.

## AUTHOR CONTRIBUTIONS

R.J.M. and P.A.F. conceived and designed research; R.J.M. performed experiments; R.J.M. and P.C. analyzed the data; R.J.M. and P.A.F. interpreted results of experiments, R.J.M. prepared figures; R.J.M. drafted manuscript; R.J.M., A.J.C., P.C., and P.A.F. edited and revised manuscript; R.J.M., A.J.C., P.C., and P.A.F. approved the final version of the manuscript.

## References

[phy214283-bib-0001] Anderson, W. D. III , Strayer, S. M. , Anderson, W. D. , Strayer, S. M. , Anderson Iii, W. D. , & Strayer, S. M. (2013). Evaluation of nausea and vomiting in adults: A case‐based Approach. American Family Physician, 88, 371–379. 24134044

[phy214283-bib-0002] Andrews, P. L. R. , & Horn, C. C. (2006). Signals for nausea and emesis: Implications for models of upper gastrointestinal diseases. Autonomic Neuroscience, 125, 100–115. 16556512 10.1016/j.autneu.2006.01.008 PMC2658708

[phy214283-bib-0003] Babic, T. , Browning, K. N. , Mcmahon, T. , Van, Z. P. C. M. , & Gilad, A. A. (2014). The role of vagal neurocircuits in the regulation of nausea and vomiting. European Journal of Pharmacology, 722, 38–47. 10.1016/j.ejphar.2013.08.047 24184670 PMC3893663

[phy214283-bib-0004] Bakdash, J. Z. , & Marusich, L. R. (2017). Repeated measures correlation. Frontiers in Psychology, 8, 1–13. 10.3389/fpsyg.2017.00456 28439244 PMC5383908

[phy214283-bib-0005] Bakdash, J. Z. , & Marusich, L. R. (2017). rmcorr: Repeated measures correlation [Online]. https://cran.r-project.org/package=rmcorr. 10.3389/fpsyg.2017.00456 PMC5383908 28439244

[phy214283-bib-0006] Berning, J. M. , Adams, K. J. , Climstein, M. , & Stamford, B. A. (2007). Metabolic demands of “junkyard” training: Pushing and pulling a motor vehicle. Journal of Strength and Conditioning Research, 21, 853–856. 17685675 10.1519/R-18335.1

[phy214283-bib-0007] Boscan, P. , Pickering, A. E. , & Paton, J. F. R. (2002). The nucleus of the solitary tract: An integrating station for nociceptive and cardiorespiratory afferents. Experimental Physiology, 87, 259–266. 11856972 10.1113/eph8702353

[phy214283-bib-0008] Brock‐Utne, J. G. , Gaffin, S. L. , Wells, M. T. , Gathiram, P. , Sohar, E. , James, M. F. , … Norman, R. J. (1988). Endotoxaemia in exhausted runners after a long‐distance race. South African Medical Journal, 73, 533–536. 3375945

[phy214283-bib-0009] Burgomaster, K. A. , Howarth, K. R. , Phillips, S. M. , Rakobowchuk, M. , MacDonald, M. J. , McGee, S. L. , & Gibala, M. J. (2008). Similar metabolic adaptations during exercise after low volume sprint interval and traditional endurance training in humans. Journal of Physiology, 586, 151–160. 10.1113/jphysiol.2007.142109 17991697 PMC2375551

[phy214283-bib-0010] Calza, L. , Manfredi, R. , & Chiodo, F. (2005). Hyperlactataemia and lactic acidosis in HIV‐ infected patients receiving antiretroviral therapy. Clinical Nutrition, 24, 5–15. 10.1016/j.clnu.2004.03.009 15681097

[phy214283-bib-0011] Chong, E. , Guelfi, K. J. , & Fournier, P. A. (2011). Effect of a carbohydrate mouth rinse on maximal sprint performance in competitive male cyclists. Journal of Science and Medicine in Sport, 14, 162–167. 10.1016/j.jsams.2010.08.003 20932798

[phy214283-bib-0012] Cohen, J. (1988). Statistical power analysis for the behavioral sciences. Hilsdale. 2nd ed Hillsdale, NJ: Lawrence Earlbaum Associates.

[phy214283-bib-0013] Costa, R. J. S. , Snipe, R. M. J. , Kitic, C. M. , & Gibson, P. R. (2017). Systematic review: Exercise‐induced gastrointestinal syndrome—Implications for health and intestinal disease. Alimentary Pharmacology & Therapeutics, 46, 246–265. 28589631 10.1111/apt.14157

[phy214283-bib-0014] de Oliveira, E. P. , & Burini, R. C. (2009). The impact of physical exercise on the gastrointestinal tract. Current Opinion in Clinical Nutrition and Metabolic Care, 12, 533–538. 10.1097/MCO.0b013e32832e6776 19535976

[phy214283-bib-0015] de Oliveira, E. P. , Burini, R. C. , & Jeukendrup, A. (2014). Gastrointestinal complaints during exercise: Prevalence, etiology, and nutritional recommendations. Sport Medicine, 44, 79–85. 10.1007/s40279-014-0153-2 PMC4008808 24791919

[phy214283-bib-0016] Dean, J. B. , Bayliss, D. A. , Erickson, J. T. , Lawing, W. L. , & Millhorn, D. E. (1990). Depolarization and stimulation of neurons in nucleus tractus solitarii by carbon dioxide does not require chemical synaptic input. Neuroscience, 36, 207–216. 10.1016/0306-4522(90)90363-9 2120613

[phy214283-bib-0017] Deighton, K. , Barry, R. , Connon, C. E. , & Stensel, D. J. (2013). Appetite, gut hormone and energy intake responses to low volume sprint interval and traditional endurance exercise. European Journal of Applied Physiology, 113, 1147–1156. 10.1007/s00421-012-2535-1 23111564

[phy214283-bib-0018] Gibala, M. J. , Little, J. P. , MacDonald, M. J. , & Hawley, J. A. (2012). Physiological adaptations to low‐volume, high‐intensity interval training in health and disease. Journal of Physiology, 590, 1077–1084. 10.1113/jphysiol.2011.224725 22289907 PMC3381816

[phy214283-bib-0019] Gillen, J. B. , Percival, M. E. , Skelly, L. E. , Martin, B. J. , Tan, R. B. , Tarnopolsky, M. A. , & Gibala, M. J. (2014). Three minutes of all‐out intermittent exercise per week increases skeletal muscle oxidative capacity and improves cardiometabolic health. PLoS ONE, 9, 1–9. 10.1371/journal.pone.0111489 PMC4218754 25365337

[phy214283-bib-0020] Gregory, N. S. , Brito, R. G. , Fusaro, M. C. G. O. , & Sluka, K. A. (2016). ASIC3 is required for development of fatigue‐induced hyperalgesia. Molecular Neurobiology, 53, 1020–1030. 10.1007/s12035-014-9055-4 25577172 PMC4499332

[phy214283-bib-0021] Grove, J. R. , & Prapavessis, H. (1992). Preliminary evidence for the reliability and validity of an abbreviated Profile of Mood States. International Journal of Sport Psychology, 23, 93–109

[phy214283-bib-0022] Hermansen, L. , & Osnes, J. B. (1972). Blood and muscle pH after maximal exercise in man. Journal of Applied Physiology, 32(3), 304–308. 10.1152/jappl.1972.32.3.304 5010039

[phy214283-bib-0023] Huda, R. , McCrimmon, D. R. , & Martina, M. (2013). pH modulation of glial glutamate transporters regulates synaptic transmission in the nucleus of the solitary tract. Journal of Neurophysiology, 110, 368–377. 10.1152/jn.01074.2012 23615553 PMC3727073

[phy214283-bib-0024] Huda, R. , Pollema‐Mays, S. L. , Chang, Z. , Alheid, G. F. , McCrimmon, D. R. , & Martina, M. (2012). Acid‐sensing ion channels contribute to chemosensitivity of breathing‐related neurons of the nucleus of the solitary tract. Journal of Physiology, 590, 4761–4775. 10.1113/jphysiol.2012.232470 22890703 PMC3487035

[phy214283-bib-0025] Immke, D. C. , & McCleskey, E. W. (2001). Lactate enhances the acid‐sensing Na+ channel on ischemia‐sensing neurons. Nature Neuroscience, 4, 869–870. 10.1038/nn0901-869 11528414

[phy214283-bib-0026] Jurovich, M. R. , Wooldridge, J. D. , & Force, R. W. (1997). Metformin‐associated nonketotic metabolic acidosis. Annals of Pharmacotherapy, 31, 53–55. 10.1177/106002809703100108 8997466

[phy214283-bib-0027] Koch, K. L. (2014). Gastric dysrhythmias: A potential objective measure of nausea. Experimental Brain Research, 232(8), 2553–2561. 10.1007/s00221-014-4007-9 24916149

[phy214283-bib-0028] Kondo, T. , Nakae, Y. , Mitsui, T. , Kagaya, M. , Matsutani, Y. , Horibe, H. , & Read, N. W. (2001). Exercise‐induced nausea is exaggerated by eating. Appetite, 36, 119–125. 10.1006/appe.2000.0391 11237347

[phy214283-bib-0029] Kraut, J. A. , & Madias, N. E. (2014). Lactic acidosis. New England Journal of Medicine, 371, 2309–2319. 10.1056/NEJMra1309483 25494270

[phy214283-bib-0030] Kucharczyk, J. (1991). Humoral factors in nausea and emesis In Kucharczyk J., Stewart D. J., & Miller A. D. (Eds.), Nausea and vomiting: Recent research and cliniical advances (pp. 59–76). Boca Raton, FL: CRC Press.

[phy214283-bib-0031] Law, L. A. F. , Sluka, K. A. , McMullen, T. , Lee, J. , Arendt‐Nielsen, L. , & Graven‐Nielsen, T. (2008). Acidic buffer induced muscle pain evokes referred pain and mechanical hyperalgesia in humans. Pain, 140, 254–264. 10.1016/j.pain.2008.08.014 18835099 PMC2613646

[phy214283-bib-0032] Leiper, J. B. , Broad, N. P. , & Maughan, R. J. (2001). Effect of intermittent high‐intensity exercise on gastric emptying in man. Medicine and Science in Sports and Exercise, 33, 1270–1278. 10.1097/00005768-200108000-00005 11474326

[phy214283-bib-0033] Levin, L. R. , & Buck, J. (2014). Physiological roles of acid‐base sensors. Annual Review of Physiology, 77, 347–362. 10.1146/annurev-physiol-021014-071821 25340964

[phy214283-bib-0034] Lin, L. , Jones, S. , & Talman, W. (2018). Cellular localization of acid‐sensing ion channel 1 in Rat nucleus tractus solitarii. Cellular and Molecular Neurobiology, 38, 219–232. 10.1007/s10571-017-0534-9 28825196 PMC11482015

[phy214283-bib-0035] MacDougall, J. D. , Hicks, A. L. , MacDonald, J. R. , McKelvie, R. S. , Green, H. J. , & Smith, K. M. (1998). Muscle performance and enzymatic adaptations to sprint interval training. Journal of Applied Physiology, 84, 2138–2142. 10.1152/jappl.1998.84.6.2138 9609810

[phy214283-bib-0036] Magistretti, P. J. , & Allaman, I. (2018). Lactate in the brain: From metabolic end‐product to signalling molecule. Nature Reviews Neuroscience, 19, 235–249. 29515192 10.1038/nrn.2018.19

[phy214283-bib-0037] Maren, T. H. (2017). Effect of varying CO_2_ equilibria on rates of HCO_3_ ^‐^ formation in cerebrospinal fluid. Journal of Applied Physiology, 47, 471–477. 10.1152/jappl.1979.47.3.471 118142

[phy214283-bib-0038] Marsh, S. (2016).Why you sometimes feel sick after exercise [Online]. Retrieved from https://coach.nine.com.au/fitness/why-you-sometimes-feel-sick-after-exercise/c37bccea-6351-46f4-98df-027bdae75c9e.

[phy214283-bib-0039] McMahon, S. , & Jenkins, D. (2002). Factors affecting the rate of phosphocreatine resynthesis following intense exercise. Sport Medicine, 32, 761–784. 10.2165/00007256-200232120-00002 12238940

[phy214283-bib-0040] Mizock, B. A. (1989). Lactic acidosis. Disease‐a‐Month, 35(4), 237–300. http://www.ncbi.nlm.nih.gov/pubmed/2656163 [15 Mar. 2018]. 10.1016/0011-5029(89)90021-7 2656163

[phy214283-bib-0041] Muth, E. R. , Stern, R. M. , Thayer, J. F. , & Koch, K. L. (1996). Assessment of the multiple dimensions of nausea: The nausea profile (NP). Journal of Psychosomatic Research, 40, 511–520. 8803860 10.1016/0022-3999(95)00638-9

[phy214283-bib-0042] Myers, J. , McAuley, P. , Lavie, C. J. , Despres, J.‐P. , Arena, R. , & Kokkinos, P. (2015). Physical activity and cardiorespiratory fitness as major markers of cardiovascular risk: Their independent and interwoven importance to health status. Progress in Cardiovascular Diseases, 57, 306–314. 25269064 10.1016/j.pcad.2014.09.011

[phy214283-bib-0043] Nattie, E. , & Li, A. (2012). Central chemoreceptors: Locations and functions. Comprehensive Physiology, 2, 221–254. 23728974 10.1002/cphy.c100083 PMC4802370

[phy214283-bib-0044] Naves, L. A. , & McCleskey, E. W. (2005). An acid‐sensing ion channel that detects ischemic pain. Brazilian Journal of Medical and Biological Research, 38, 1561–1569. 10.1590/S0100-879X2005001100001 16258623

[phy214283-bib-0045] O’Grady, G. , Wang, T.‐H.‐H. , Du, P. , Angeli, T. , Lammers, W. J. E. P. , & Cheng, L. K. (2014). Recent progress in gastric arrhythmia: Pathophysiology, clinical significance and future horizons. Clinical and Experimental Pharmacology and Physiology, 41, 854–862. 25115692 10.1111/1440-1681.12288 PMC4359928

[phy214283-bib-0046] Oman, C. M. (2012). Are evolutionary hypotheses for motion sickness" just‐so" stories? Journal of Vestibular Research, 22, 117–127. 23000611 10.3233/VES-2011-0432

[phy214283-bib-0047] Price, C. J. , Hoyda, T. D. , & Ferguson, A. V. (2008). The area postrema: A brain monitor and integrator of systemic autonomic state. Neuroscientist, 14, 182–194. 18079557 10.1177/1073858407311100

[phy214283-bib-0048] Putnam, R. W. , Filosa, J. A. , & Ritucci, N. A. (2004). Cellular mechanisms involved in CO_2_ and acid signaling in chemosensitive neurons. American Journal of Physiology, 287, C1493–C1526. 15525685 10.1152/ajpcell.00282.2004

[phy214283-bib-0049] Qamar, M. A. , & Read, A. E. (1987). Effects of exercise on mesenteric blood flow in man. Gut, 28, 583–587. 10.1136/gut.28.5.583 3596339 PMC1432887

[phy214283-bib-0050] Quigley, E. M. M. M. , Hasler, W. L. , & Parkman, H. P. (2001). AGA technical review on nausea and vomiting. Gastroenterology, 120, 263–286. 10.1053/gast.2001.20516 11208736

[phy214283-bib-0051] R Core Team . (2017). A language and environment for statistical computing [Online]. R Foundation for Statistical Computing https://www.r-project.org/.

[phy214283-bib-0052] Richards, J. C. , Johnson, T. K. , Kuzma, J. N. , Lonac, M. C. , Schweder, M. M. , Voyles, W. F. , & Bell, C. (2010). Short‐term sprint interval training increases insulin sensitivity in healthy adults but does not affect the thermogenic response to Î^2^‐adrenergic stimulation. Journal of Physiology, 588, 2961–2972. 20547683 10.1113/jphysiol.2010.189886 PMC2956910

[phy214283-bib-0053] Rundqvist, H. C. , Esbjörnsson, M. , Rooyackers, O. , Österlund, T. , Moberg, M. , Apro, W. , … Jansson, E. (2017). Influence of nutrient ingestion on amino acid transporters and protein synthesis in human skeletal muscle after sprint exercise. Journal of Applied Physiology, 123, 1501–1515. 10.1152/japplphysiol.00244.2017 28860165

[phy214283-bib-0054] Samborski, P. , Chmielarz‐Czarnocińska, A. , & Grzymisławski, M. (2013). Exercise‐induced vomiting. Przegląd Gastroenterologiczny, 8, 396–400. 10.5114/pg.2013.39924 PMC4027831 24868290

[phy214283-bib-0055] SAS Intitute . (2018). The SAS system for Windows. Cary, NC: SAS Institue Inc.

[phy214283-bib-0056] Schmidt, N. B. (1999). Examination of differential anxiety sensitivities in panic disorder: a test of anxiety sensitivity subdomains predicting fearful responding to a 35% CO2 challenge [Online]. Cognitive Therapy and Research, 23, 3–19. https://link.springer.com/content/pdf/10.1023/A:1018754522981.pdf [7 Jun. 2019].

[phy214283-bib-0057] Schrank, B. , Schoser, B. , Klopstock, T. , Schneiderat, P. , & Horvath, R. (2017). Lifetime exercise intolerance with lactic acidosis as key manifestation of novel compound heterozygous ACAD9 mutations causing complex I deficiency. Neuromuscular Disorders, 27, 473–476. 10.1016/j.nmd.2017.02.005 28279569

[phy214283-bib-0058] Sharma, S. , & Aggarwal, S. Hyperchloremic acidosis [Online]. StatPearls Publishing http://www.ncbi.nlm.nih.gov/pubmed/29493965 [9 Apr. 2018]. 29493965

[phy214283-bib-0059] Singh, P. , Yoon, S. S. , & Kuo, B. (2016). Nausea: A review of pathophysiology and therapeutics. Therapeutic Advances in Gastroenterology, 9, 98–112. 26770271 10.1177/1756283X15618131 PMC4699282

[phy214283-bib-0060] Stern, R. M. , Koch, K. L. , & Andrews, P. (2011). Nausea: Mechanisms and management. Cary, NC: Oxford University Press.

[phy214283-bib-0061] Stuempfle, K. J. , Valentino, T. , Hew‐Butler, T. , Hecht, F. M. , & Hoffman, M. D. (2016). Nausea is associated with endotoxemia during a 161‐km ultramarathon. Journal of Sports Sciences, 34, 1662–1668. 10.1080/02640414.2015.1130238 26707127

[phy214283-bib-0062] Tournadre, J. P. , Allaouchiche, B. , Malbert, C. H. , & Chassard, D. (2000). Metabolic acidosis and respiratory acidosis impair gastro‐pyloric motility in anesthetized pigs [Online]. Anesthesia and Analgesia, 90, 74–79. http://www.ncbi.nlm.nih.gov/pubmed/10624982. 10.1097/00000539-200001000-00018 10624982

[phy214283-bib-0063] Treisman, M. (1977). Motion sickness: An evolutionary hypothesis. Science (80‐), 197, 493–495. 10.1126/science.301659 301659

[phy214283-bib-0064] Tresguerres, M. , Buck, J. , & Levin, L. R. (2010). Physiological carbon dioxide, bicarbonate, and pH sensing. Pflügers Archiv ‐ European Journal of Physiology, 460, 953–964. 10.1007/s00424-010-0865-6 20683624 PMC2967379

[phy214283-bib-0065] West, B. T. , Welch, K. B. , & Galecki, A. T. (2014). Linear mixed models: A practical guide using statistical software. Boca Raton, Florida: CRC Press.

[phy214283-bib-0066] Williams, C. B. , Zelt, J. G. E. , Castellani, L. N. , Little, J. P. , Jung, M. E. , Wright, D. C. , … Gurd, B. J. (2013). Changes in mechanisms proposed to mediate fat loss following an acute bout of high‐intensity interval and endurance exercise. Applied Physiology, Nutrition and Metabolism, 38, 1236–1244. 10.1139/apnm-2013-0101 24195624

[phy214283-bib-0067] Yagi, J. , Wenk, H. N. , Naves, L. A. , & McCleskey, E. W. (2006). Sustained currents through ASIC3 ion channels at the modest pH changes that occur during myocardial ischemia. Circulation Research, 99, 501–509. 10.1161/01.RES.0000238388.79295.4c 16873722

